# Report of the Committee of the American Surgical Association on the Medicolegal Relations of the X-Rays

**Published:** 1900-09

**Authors:** J. William White

**Affiliations:** Philadelphia, Chairman


### REPORT OF THE COMMITTEE OF THE AMERICAN SURGICAL ASSOCIATION ON THE MEDICO-LEGAL RELATIONS OF THE X-RAYS.

By J. WILLIAM WHITE, M. D., of Philadelphia, Chairman.

[*American Journal of Medical Sciences*, July, 1900.]

CONCLUSIONS.

1. The routine employment of the *x*-ray in cases of fracture is not at present of sufficient definite advantage to justify the teaching that it should be used in every case. If the surgeon is in doubt as to his diagnosis, he should make use of this as of every other available means to add to his knowledge of the case, but even then he should not forget the grave possibilities of misinterpretation. There is evidence that in competent hands plates may be made that will fail to reveal the presence of existing fractures or will appear to show a fracture that does not exist.

2. In the regions of the base of the skull, the spine, the pelvis, and the hips, the *x*-ray results have not as yet been thoroughly satisfactory, although good skiagraphs have been made of lesions in the last three localities. On account of the rarity of such skiagraphs of these parts special caution should be observed, when they are affected, in basing upon *x*-ray testimony any important diagnosis or line of treatment.

3. As to questions of deformity, skiagraphs alone, without expert surgical interpretation, are generally useless and frequently misleading. The appearance of deformity may be produced in any normal bone, and existing deformity may be grossly exaggerated.

4. It is not possible to distinguish after recent fractures between cases in which perfectly satisfactory callus has formed and cases which will go on to non-union. Neither can fibrous union be distinguished from union by callus in which lime-salts have not yet been deposited. There is abundant evidence to show that the use of the *x*-ray in these cases should be regarded as merely the adjunct to other surgical methods, and that its testimony is especially fallible.

5. The evidence as to *x*-ray burns seems to show that in the majority of cases they are easily and certainly preventable. The essential cause is still a matter of dispute. It seems not unlikely when the strange susceptibilities due to idiosyncrasy are remembered that in a small number of cases it may make a given individual especially liable to this form of injury.

6. In the recognition of foreign bodies the skiagraph is of the very greatest value; in their localisation it has occasionally failed. The mistakes recorded in the former case should easily have been avoided; in the latter they are becoming less and less frequent, and by the employment of accurate mathematical methods can probably in time be eliminated. In the meanwhile, however, the surgeon who bases an important operation on the localisation of a foreign body buried in the tissues should remember the possibility of error that still exists.

7. It has not seemed worth while to attempt a review of the situation from the strictly legal stand-point. It would vary in different states and with different judges to interpret the law. The evidence shows, however, that in many places and under many differing circumstances the skiagraph will undoubtedly be a factor in medico-legal cases.

8. The technicalities of its production, the manipulation of the apparatus, etc., are already in the hands of specialists, and with that subject also it has not seemed worth while to deal. But it is earnestly recommened that the surgeon should so familiarise himself with the appearance of skiagraphs, with their distortions, with the relative values of their shadows and outlines, as to be himself the judge of their teachings, and not depend upon the interpretation of others who may lack the wide experience with surgical injury and disease necessary for the correct reading of these pictures.

[These conclusions were unanimously adopted as expresssing the views of the American Surgical Association.]

---

The Opium Habit in the East.—The extent to which the opium habit prevails amongst Europeans in the East is only properly appreciated by those specially brought into contact with the victims; they will be interested in the announcement made by Dr. Neil McLeod, of Shanghai, that it is possible to cure the habit by the administration of sodium bromide. He gives the drug "in two doses of two drachms, in solution, every two hours for the first two days, and one drachm on the third day," and adds that "three ounces of the drug in all will probably suffice in most cases." The treatment is quite safe and is well worth trying.—*Journal of Tropical Medicine*,

